# Current Approaches Targeting the Wound Healing Phases to Attenuate Fibrosis and Scarring

**DOI:** 10.3390/ijms21031105

**Published:** 2020-02-07

**Authors:** Amina El Ayadi, Jayson W. Jay, Anesh Prasai

**Affiliations:** Burn, Trauma, and Critical Care Research Labs, Department of Surgery, University of Texas Medical Branch, Galveston, TX 77550, USA; jwjay@utmb.edu (J.W.J.); anprasai@utmb.edu (A.P.)

**Keywords:** fibrosis, wound healing, burn, hypertrophic scarring, myofibroblasts, macrophages, inflammation, EMT

## Abstract

Cutaneous fibrosis results from suboptimal wound healing following significant tissue injury such as severe burns, trauma, and major surgeries. Pathologic skin fibrosis results in scars that are disfiguring, limit normal movement, and prevent patient recovery and reintegration into society. While various therapeutic strategies have been used to accelerate wound healing and decrease the incidence of scarring, recent studies have targeted the molecular regulators of each phase of wound healing, including the inflammatory, proliferative, and remodeling phases. Here, we reviewed the most recent literature elucidating molecular pathways that can be targeted to reduce fibrosis with a particular focus on post-burn scarring. Current research targeting inflammatory mediators, the epithelial to mesenchymal transition, and regulators of myofibroblast differentiation shows promising results. However, a multimodal approach addressing all three phases of wound healing may provide the best therapeutic outcome.

## 1. Introduction

**Figure 1:**
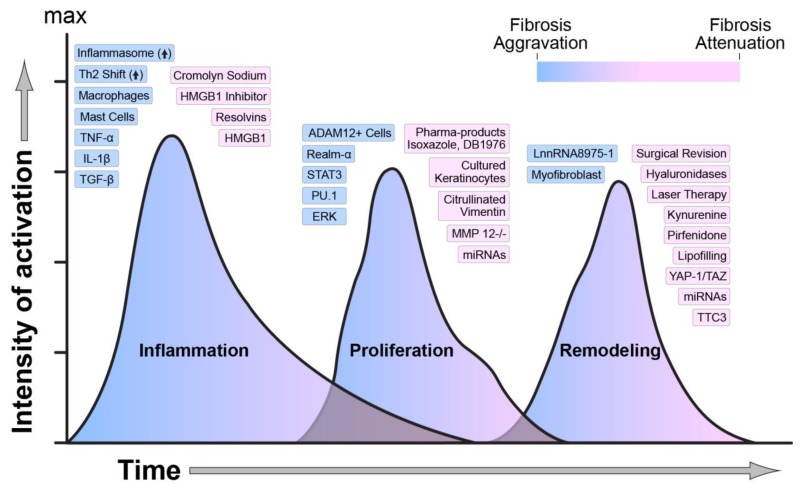
Regulators of wound healing and scarring. The temporal activation, overlap, and intensity of activation of each phase of wound healing are regulated by numerous molecular, biological, and mechanical factors. The figure indicates how each one of these factors is modulating wound healing towards an aggravation or an attenuation of fibrosis. Blue indicates activation, and pink indicates attenuation of fibrosis.

Fibrosis is a natural process to restore tissue function during healthy wound healing. When pathological, fibrosis can result in detrimental scarring and dysfunctional tissue. These scars result from increased and prolonged inflammation, leading to suboptimal wound healing. In burns, the hypermetabolic stress response manifests as a sustained surge of inflammatory cytokines [1] that affect the migration of immune cells, including neutrophils and macrophages, to the wound area. Tissue damage following burn induces the activation of various damage-associated molecules (DAMPs) that signal immune cells to migrate to the wound. Initial inflammation activates multiple cell populations in the dermis and epidermis to aid in wound re-epithelization and closure [2]. Among the inflammatory cytokines, transforming growth factor-beta (TGF-β) enables differentiation of fibroblasts to myofibroblasts to assist with wound coverage. However, the persistent activation of TGF-β signaling provides an erroneous signal to myofibroblasts for continuous extracellular matrix (ECM) production leading to pathological scarring [3]. Importantly, almost all fibrotic diseases exhibit one underlying phenomenon—disarray of three distinct but overlapping phases of wound healing. The molecular mediators regulating the wound healing phases are summarized in Figure 1. While various therapeutic approaches are used to target different stages of wound healing to prevent scar development, none of these therapies have been entirely successful. This review recapitulates the most recent advancements to study the underlying mechanism of fibrosis. We analyzed the current literature on the role of various inflammatory markers in triggering the immune response following injury to protect against infections and begin the healing process. We dissected the role of epigenetics and transcriptional regulation in the expression or repression of fibrosis genes. We also analyzed the current knowledge of the mechanisms underlying myofibroblasts differentiation. Finally, we report ongoing clinical trials aiming to treat pathologic fibrosis utilizing biological and pharmacological tools.

## 2. Interacting Wound Healing Phases

It is recognized that increased and sustained inflammation leads to suboptimal wound healing and subsequent fibrotic development. Independent of the cause of injury, wound healing is characterized by three established phases that are synchronized in time and activity until complete wound closure [4]. The three stages are namely the inflammatory, proliferation, and remodeling phase [5]. Current studies have updated the roles of numerous molecular pathways in regulating each phase of wound healing, as discussed in this review.

## 3. Inflammation in the Healing Wound

In adults, tissue damage prompts the inflammatory phase of wound healing and is classically defined with the temporal activation and recruitment of various cells of innate and adaptive immunity [4]. Inflammation involves robust mechanisms allowing tissues to repair the damage, regenerate, and ultimately heal to restore function. However, not all inflammatory processes inevitably result in fibrotic pathophysiology. It is well documented that most fetal tissues heal without fibrosis, and various investigations have sought to elucidate this scarless fetal phenotype [6,7,8].

It was previously thought that the amniotic environment within the mammalian placenta was beneficial for scarless healing. The intrauterine setting is a naturally sterile compartment bathed in abundant growth factors, is free of pathogens, and lacks stimuli to initiate inflammation; thus, tissues were hypothesized to heal without fibrosis. Indeed, initial investigations demonstrated scarless fetal wound healing in several amniote species, including rats, mice, pigs, sheep, and humans [6,7,9]. These studies showed inherent tissue structure differences and environmental molecular variances between fetal and adult wounds that ultimately determined scarless healing capacity [10,11,12,13]. Significantly, experiments demonstrated that adult skin, when placed in a fetal environment and then subsequently wounded, healed with scar formation [14], underscoring the intrinsic differences between adult and fetal wound healing. As investigations into scarless pathophysiology evolved, data demonstrated that fetal wound healing is also a function of wound defect size and gestational age; larger defect size or more advanced gestation age resulted in a higher probability of scar formation [8,15]. Gestational age was significant because it demonstrated that immune system maturation allows for a more robust response and eventually results in some level of fibrosis, further supporting the necessity of the inflammatory response in fibrotic pathogenesis. However, to initiate inflammatory reactions, factors from tissue damage must first be present.

### 3.1. Inflammation Arises Due to Tissue Damage

Tissue damage results both in necrotic and apoptotic cell death, in turn releasing direct factors and chemoattractant molecules that initiate the recruitment of immune cells. Traumatic injuries, such as deep thermal burns, along with other ischemic, toxic, or allergic insults, result in cell death culminating in the release of inflammatory stimuli derived from cytosolic proteins and various organelles including nuclear and mitochondrial nucleic acids [1,16]. Collectively, these are known as danger-associated molecular patterns (DAMPs), which in turn bind to their associated pattern-recognition receptors (PRRs), recruiting immune cells and driving initial inflammation [17]. DAMPs are one of the primary signaling molecules that activate the immune system to prevent infection of injured tissue [17].

The high-mobility group box-1 protein (HMGB1) is one of the first DAMP proteins described and as such, one of the best-characterized. Interestingly, HMGB1 has been shown to follow secretory pathways by actively stressed cells as well [2,18]. By adjusting oxidation states, HMGB1 binds to specific PRRs in adjacent cells, further directing exclusive signaling, immune cell recruitment, and may have direct impacts on fibrogenesis. Studies have demonstrated that HMGB1 acts as a chemoattractant for monocytes and that HMGB1 signaling can reprogram tissue macrophages to a more profibrotic M1 phenotype, which is further discussed in Section 4.2 [19,20]. Other investigations show that HMGB1 can bind to the receptor for advanced glycation end products (RAGE), activating a fibrotic signaling cascade and directly upregulating collagen production [21]. Through exclusionary signaling and inflammatory polarization, HMGB1 has been suggested as a biomarker in active fibrotic diseases, including hepatic and cutaneous fibrosis [22,23,24]. As such an essential inflammatory factor linked to fibrogenesis, several studies have shown pharmacological HMGB1 inhibition to limit fibrosis in a rat model of non-alcoholic steatohepatitis and cardiac fibrosis [25,26]. This evidence further suggests that HMGB1 may become a critical target to prevent pathological scarring in the future.

Another interesting phenomenon involving the neutrophil cell population can occur during the initial inflammatory phase in many pathologies. Neutrophils are one of the first cell populations found in damaged tissue. Classically, neutrophils function to engulf potential pathogens or secrete anti-microbial proteins to prevent wound infection. However, a third neutrophil function has been elucidated over the past decade describing a unique cellular process that actively emits nuclear material to signal other inflammatory cells and sequester pathogens. The extruded nuclear material contains higher-order chromatin that clumps together forming fibrous strand meshes resembling a net and thus came to be known as neutrophil extracellular traps (NETs). NET formation is exclusive to neutrophils and separate from necrotic or apoptotic chromatin release; therefore, the extraordinary process was termed NETosis. Uncondensed nucleosome complexes released during NETosis include histone cores with associated dsDNA. NETs formed during this inflammatory process have also been linked to fibrotic pathologies. Increased NET formation has been observed in acute kidney injury (AKI) in humans; investigators showed that by enhancing (endothelial cell) tubular necrosis, NETs further exacerbated AKI fibrotic progression [27]. Another recent investigation has shown enhanced NET formation in the blood of cystic fibrosis patients that was precipitated by aberrant and delayed neutrophil apoptosis [28], further linking this unique process to fibrotic pathogenesis. Additionally, individual histones may release from NETs and themselves serve as robust DAMPs that vigorously protract excessive inflammation. Several studies have shown histones to interact with toll-like receptors that activate pro-inflammatory signaling and have proved cytotoxic to endothelial cells and myocytes [29,30]. Furthermore, it has been demonstrated that the acute inflammatory phase C-reactive protein can limit the cytotoxicity of circulating free histones through competitive inhibition of lysosome binding [31], lending further insight into histone DAMP signaling. These studies all suggest that limiting NETosis during inflammatory processes could have the potential to abrogate subsequent fibrosis as well.

Damaged tissues also release nuclear and mitochondrial nucleic acids through necrosis and serve as potent DAMPs that illicit the inflammatory response, activating immune cells and leading to the production of pro-inflammatory cytokines including potent fibrogenic initiators interleukin-1beta (IL-1β), tumor necrosis factor-alpha (TNF-α), and transforming growth factor-beta (TGF-β), which further direct the adaptive immune response [32,33,34,35]. In recent years, studies have demonstrated that extracellular DNA fragments, either free or contained in extracellular vesicles, are phagocytized by tissue macrophages. Once phagocytized, DNA can stimulate the intracellular multiprotein complex receptor of innate immunity known as the inflammasome through interaction with AIM2 (absent in melanoma 2), an accessory protein required for inflammasome function [36,37]. Inflammasome activation is known to upregulate the production of pro-inflammatory IL-1β, IL-18, and TGF-β cytokines and induces fibrotic signaling [38,39,40]. This link to fibrosis further suggests that targeting inflammasome activation during the inflammatory phase could be a potential therapy to reduce fibrogenesis and warrants further investigation.

Additionally, damaged endothelium releases complement, which is a potent chemoattractant for neutrophils. Infiltrating neutrophils substantially increase the necrotic load and further contribute to a pro-inflammatory cycle through TNF-α and IL-1α/β production. Other factors known as alarmins are released from stressed and irreparably damaged cells following tissue insult and contribute directly to immune cell migration to the wound [34,41]. By DAMP production and through alarmin release, tissue damage ultimately results in the recruitment and activation of inflammatory cells tasked with the primary goal to moderate damage and begin the repair process.

### 3.2. Inflammatory Progression

IL-25 and IL-33 are inflammatory alarmins released by damaged epithelial cells and have been shown to induce a Th2 shift in CD4^+^ populations, which in turn is thought to promote fibrogenesis [41,42,43]. Representative Th2 cytokines include IL-4, IL-5, IL-10, and IL-13. IL-4 and IL-13 have overlapping signaling and are known to promote collagen production via STAT pathways [44,45] and produce significantly higher expression of extracellular matrix proteins in several fibrotic pathologies [46,47,48,49]. Burns resulting in severe fibrosis have also been shown to promote a Th2 shift and concomitantly increased Th2 cytokines [50,51]. Conversely, these same profibrotic Th2 cytokines inhibit the expression of many pro-inflammatory mediators, including TNF-α, IL-1, and IL-6, all known fibrogenic initiators, meaning Th2 pathway inhibition necessarily upregulates pro-inflammatory Th1 profiles. Ideally, CD4^+^ subsets must be balanced for an adequate inflammatory response and recruitment of other immune cell populations.

Mast cells may be another vital, yet overlooked, immune cell population needed as critical effectors in wound healing processes. Apart from their known role in allergy and anaphylaxis, several investigations have now shown mast cells’ growing importance in fibrogenesis and progression. Mast cells exert their immune influence through a process of regulated exocytosis known as degranulation. Following degranulation, mast cells release preformed mediators such as histamine that contribute directly to vasodilation and vascular permeability during inflammation. Histamine metabolites have been found significantly elevated in adult burn patients [52,53], and the multifaceted vasoactive amine has been shown to contribute to myofibroblast conversion with subsequent upregulation of collagen and other ECM components [54,55]. Histamine inhibition was also shown to mitigate fibrotic progression in mouse models of fibrotic cholangitis, underscoring its fibrogenic capacity [56,57]. Secreted mast cell serine proteases such as tryptase and chymase as well are known to upregulate collagen production and stimulate fibroblast proliferation and myofibroblast differentiation [58,59,60]. Our research group has also shown significantly increased mast cell densities in hypertrophic scars (HS) of severely burned pediatric survivors as well as burn-injured red Duroc pigs [61,62]. Furthermore, pharmacologic mast cell stabilization through topical application of cromolyn sodium or systemic administration of ketotifen can mitigate fibrotic progression in vivo [63,64,65]. The current data demonstrate the importance of mast cells during inflammation and highlight this versatile cell’s fibrotic capabilities. Despite the plethora of cellular and molecular participants described, resolving inflammation is critical to continuing the wound healing cascade.

Natural killer (NK) cells act as a liaison between the innate and adaptive immune systems. These cells sense stressed cells via their CD944/NKG2A receptors in humans and play an active role in tissue homeostasis following injury. The role of natural killer T (NKT) cells as negative regulators of wound healing has been extensively reviewed [4,66]. The absence of NKT cells in a mouse model of excisional wound injury resulted in accelerated wound healing and decreased risk of infection [67], while in wild type animals, NKT cells were expressed in the early phase of wound healing and regulated other immune cells including neutrophils and macrophages in addition to TGF-b secretion and collagen deposition [67]. Tanno et al. also reported that Jα18KO mice deficient in invariant NKT (iNKT) displayed a delay in wound closure by decreasing collagen deposition, myofibroblasts differentiation, and angiogenesis [68]. The same group has shown that iNKT cells promote skin wound healing by reducing the prolonged inflammatory response after injury [69]. Besides wound healing, these cells play an active role in other skin diseases like atopic dermatitis [70]. Taken together, these studies demonstrate that natural killer cells play an active role in all phases of wound healing including inflammation, proliferation, and tissue remodeling.

### 3.3. Resolving Inflammation to Initiate the Proliferation Phase of Wound Healing

Resolution is the final and essential stage of the wound healing inflammatory cascade. It is an active process directed by several factors designed to provide apoptotic or chemotactic stimuli to inflammatory cells that ultimately shifts progress toward the proliferation phase. Numerous mediators have been identified to control resolution; among the classes of factors best described are the resolvins (Rv). Rvs are bioactive lipid compounds derived from the arachidonic acid metabolic pathway and, as their name implies, are rapidly generated during inflammation resolution [71]. Phenotypic anti-inflammatory macrophages show upregulation of multiple RVs that have been shown to inhibit neutrophil chemotaxis and prevent entry into wound sites via cytoskeletal perturbation, and importantly, dysregulation of their resolving function protracts the inflammatory phase, further promoting fibrotic progression [72,73]. Thus, recent studies have found that exogenous application of Rvs, specifically the RvD1 lipid mediator, can significantly limit inflammation and prevent a fibrotic phenotype in both in vivo mouse models and in vitro human models of inflammatory disease [74,75,76]. Clinically, significantly higher Rv gene expression is seen after 28 days in trauma patients with uncomplicated recoveries; conversely, lower Rv gene expression was measured in complicated traumatic recoveries [77]. Similar results have also been found following severe burns. Recently, Inoue and colleagues showed postburn hepatic and renal damage was significantly reduced following intravenous administration of RvD2 [78]. Correspondingly, Bohr and colleagues [79] demonstrated RvD2 administration could prevent thrombosis following a severe burn in rats. As deep thermal injuries typically result in painful fibrosis and hypertrophic scarring, further investigations into Rv therapies are warranted.

Regulating pro- and anti-inflammatory conditions are vitally important in the healing wound, and failure of mechanisms to equilibrate this delicate balance may result in a prolonged and chronic inflammatory state, ultimately leading to fibrotic pathology. However, inflammation is a necessary process to heal damaged tissue. Limiting its detrimental impacts and resolving inflammation to initiate the proliferation phase of wound healing should be a significant research focus moving forward.

## 4. Proliferation in the Healing Wound

The dynamic progression from the inflammatory phase to the proliferative phase is one of the vital steps of wound healing. Accumulating evidence shows that prolongation or delay in the inflammatory phase has adverse effects on the remaining stages of wound healing, resulting in fibrosis [5,80]. The increase in specific cytokines and growth factors during initial inflammation drives the proliferation phase. However, prolongation in the inflammatory phase gives rise to fibrotic tissues that can manifest as cancers in some organs [81] and hypertrophic scars in the skin [82,83]. Thus, the inflammatory phase is tightly controlled and connected at cellular and molecular levels with the proliferative phase of wound healing.

The following sections discuss current understanding of the role of vital cells involved in the proliferation phase and highlight recent advancements made at pharmacological and biological levels as well as interventions that modulate these cells for a favorable wound healing scenario. Following skin injury, the platelet-rich plasma (PRP)-derived growth factor matrix provides an ideal platform for vital cells like keratinocytes, endothelial cells, fibroblasts, epidermal stem cells, and macrophages to begin proliferating, giving rise to immature granulation tissue [84,85]. The granulation tissue in the wound bed is vital to wound closure and is filled with new capillaries and connective tissue. The new capillaries and endothelial cells help with the formation of new blood vessels also referred to as angiogenesis. This process ensures that nutrients are supplied to the new granulation tissue.

### 4.1. Keratinocytes

Keratinocytes are the major cells of the epidermis. These cells produce structural and biologically active proteins and peptides that protect against various pathogens [86]. As the most peripheral cells of the skin, keratinocytes are one of the first cell populations to respond to a skin injury [87]. Over the last two decades, many therapeutic interventions have been investigated to modulate the proliferation of keratinocytes and expedite wound healing after burn. Cultured keratinocytes have evolved over the previous three decades from sheets [88], to inlay within different tissue-engineered products [89], to spray systems [90]. These methods showed promising results to accelerate wound healing and decrease fibrosis but have inherent drawbacks, with graft rejection being the most prominent [91]. Pharmacological stimulators like glycitin and phytochemical, 4′,6,7-trimethoxy-isoflavone (TMF) [92], icariin [93], and epigallocatechin-3-gallate [94] improved wound healing in different disease models by activating keratinocytes and fibroblasts in vitro and in vivo. Similarly, pharmacological activation of the Nrf2–IL-36γ pathway accelerated keratinocyte proliferation and expression of keratinocyte mitogens in fibroblasts, paving a new mechanistic path for keratinocyte modulation [95].

MicroRNA (miRNA) modulation was shown to regulate keratinocytes in various pathological scenarios. Overexpression of novel miRNA (seq-915_x4024) in keratinocytes improved skin regeneration and reduced scar formation by targeting TGF-β1/Smad signaling in a full-thickness excision mouse model [96]. Similarly, miR-132 is shown to regulate the transition from the inflammatory to the proliferative phase by dampening inflammation via STAT3 and ERK signaling as well as by activating the proliferation of keratinocytes [97]. Silencing miR-132 exacerbated inflammation, prolonged wound healing, and decreased keratinocyte proliferation in mouse and human ex vivo wound models [97]. However, miR-181b inhibited the proliferation of keratinocytes in psoriasis model via the TLR4 pathway [98].

### 4.2. Macrophages

As myeloid progenitors, macrophages are expanded in the liver and then distributed almost throughout the entire body as mature tissue-resident macrophages expressing the surface proteins F4/80 [99,100,101]. Macrophages, located at the basal and supra-basal layer of skin where they maintain tissue homeostasis, are commonly known as the Langerhans cells (LC) [102]. Following an acute cutaneous injury, neutrophils, tissue-resident macrophages, and hematopoietic monocytes arrive at the wound site by following the chemotactic gradient where they are predominantly activated via endotoxins secreted by bacteria, growth factors, or by interferon-gamma secreted by fibroblasts and natural killer cells [103]. PAMPs and DAMPs generated by damaged tissues also attract immune cells to the wound site. Macrophages are among the few active immune cells that are found from the beginning to the end of wound healing; thus, their intensity of activation, differentiation, and clearance defines the outcome of the wound [104]. As one of the most plastic cells of hematopoietic origin, macrophages constitute more than half of the cell population in granulation tissue [105]. Macrophages have significant roles in the development of fibrosis in many disease pathologies [106]. While macrophage presence and activity in the wound is vital, over-activation is pathologic as they prolong the proliferation and remodeling phases of wound healing, resulting in fibrotic tissue formation.

An increasing number of pharmacological and biological therapeutic agents have been developed to modulate macrophage polarization for improved wound healing outcomes. Macrophages are the primary producers of matrix metalloproteinases (MMPs), which have a significant role in creating a platform for new cells to grow and expand, while also transforming excess ECM during the remodeling phase [107]. MMP-12, a major macrophage-secreted elastase, has been linked to a fibrotic phenotype during the proliferation phase. Interestingly, MMP-12^-/-^ mice show decreased expression of profibrotic markers like pSMAD2, TGF-β1, PDGF-BB in the skin and liver [108]. Contrarily, MMP-10^-/-^ mice demonstrated that MMP-10 directs collagen production via macrophage modulation of MMP-13, further highlighting the pleiotropic effects of MMPs in wound healing [109].

Knipper and colleagues reported that interleukin-4 receptor α (IL-4Rα) is vital for macrophage activation and maturation, and plays an active role in tissue repair and the formation of granulation tissue [110]. IL-4Rα also promotes a pro-fibrotic phenotype by modulating collagen fibril compilation [110]. Relm-α was identified as an important mediator of IL-4Rα in macrophages as well. Macrophage activation resulted in a persistent production of lysyl hydroxylase 2 (LH2) in fibroblasts, a condition observed in aberrant human skin fibrosis [110].

Various modulators of macrophage behavior have shown promising results in improving systemic skin sclerosis (SSc), which is defined by aberrant fibrosis and vascular abnormalities. Among these modulators is the adipokine visfatin [111], the Friend leukemia virus integration 1 (Fli1) [112], and citrullinated vimentin [113]. Uderhardt and colleagues described a novel macrophage mechanism termed “cloaking” [114]. Cloaking is a process where tissue-resident macrophages respond to injury by extending their membranes to surround the wound, thus preventing tissue neutrophils from infiltrating the wound. Cloaking was not observed in macrophage-depleted animals resulting in compromised homeostasis and extensive tissue damage [114].

In our studies, we observed a significant increase in M2 phenotype macrophages in skin and scar biopsies obtained from burn survivors compared to controls [115]. Based on their secretory profile, surface antigen presentation, and differential temporal presence in the wound, macrophages are categorized into two phenotypes: M1 and M2 (Table 1).

Rather than a research focus to diminish macrophage presence in a healing wound, molecular targeting, cellular reprogramming, or modulating macrophages via pharmacological or biological mediators hold a better promise to prevent fibrosis following tissue injury.

### 4.3. Endothelial Cells

Aberrant and excessive microvessel formation is one of the salient features of fibrosis [116]. Tissue injury is generally followed by the transient loss of vascular structures resulting in a hypoxic state and activation of hypoxia-inducible factor-1 (HIF-1) signaling. The hypoxic environment further intensifies angiogenesis to satisfy the increased oxygen demand, thus promoting endothelial cell proliferation [117]. The newly formed vessels are irregular and dysfunctional, which further exacerbates inflammation. Several studies have targeted the endothelial cell population for better therapeutic outcomes in fibrotic disease conditions. Gao et al. [118] reported that leucine-rich-alpha-2-glycoprotein 1 (LRG-1) mediated biomechanical forces to improve angiogenesis. Overexpression of LRG-1 was reported in hypertrophic scars, and depletion of LRG-1 in a mice model inhibited active angiogenesis and lessened skin fibrosis [118]. Using Cdh5-creERt2/ROSA-YFP mice for vascular-specific tracking, Patel and colleagues [119] concluded that the loss of notch signaling in the transgenic mice resulted in the rapid transition of endothelial cells to mesenchymal cells (EndMT), which in turn increased fibrosis, stimulated scar tissue formation, and delayed wound healing. Xu et al. [120] used Cdc42 deficient mice to determine the role of macrophages in wound healing. Cdc42 is a Rho family protein that plays a role in microvasculature permeability. They showed that knockdown of Cdc42 significantly increases the macrophage population, induces fibrosis, and substantially delays wound healing [120]. Dulauroy et al. [121] reported that the anchored membrane metalloprotease ADAM12 expression is upregulated during skin and muscle injury. Expression of ADAM12 during injury resulted in a profibrotic state with increased interstitial collagen deposition, while genetic ablation of ADAM12 expressing cells resulted in significantly less fibrosis and better wound healing. Aberrant endothelial cell proliferation and distribution is one of the hallmarks of fibrosis, and effectively targeting molecular pathways to reduce or inhibit the expression of endothelial cells following injury holds a promising future in attenuating fibrosis.

### 4.4. Mechanosensors in Fibrosis

Dynamic and positive mechanosensory feedback mechanisms drive cells towards a profibrotic phenotype by regulating the cell’s behavior and transforming the mechanical forces into biochemical signals, with the outcome being excess ECM accumulation [122,123,124]. The harmonic combination of three main physical forces, including pressure [125], stretch/strain [125], and shear [126] holds fibroblasts together in a three-dimensional structure composed of specific ratios of various ECM components. Injury disrupts the composition of the ECM, and the ratio of type III/type I collagen increases during scar development [127]. In controlled preclinical models of hypertrophic scarring, the application of pressure garment therapy improves physical appearance and reduces the total collagen content in scars by more than 50%, while the transcript of both type I and III collagen decreased by more than 40-fold [128]. Velasquez and colleagues [129] reported that the small molecule isoxazole modulates myocardin-related transcription factors (MRTFs) to regulate cellular cytoskeleton dynamics. Isoxazole accelerated wound closure and attenuated inflammation via MRTF pathway activation. These studies suggest that interventions to modulate mechanosensory mechanisms can potentially decrease fibrosis and scarring.

### 4.5. Fibroblast Role in Fibrosis

Regardless of the type of tissue injury, fibroblasts are of mesenchymal origin and play a significant role in maintaining tissue integrity by proliferating [130], differentiating [131], and collaborating with other cells [132] both in homeostasis and disease states. They modulate fibrosis by producing the components of the ECM. In the skin, fibroblasts primarily respond to injury by covering the insult with a granulation tissue during the proliferation phase and by differentiating into a myofibroblast phenotype during the remodeling phase, driven primarily by TGF-β/Smad signaling, which is further discussed in Section 5.2 of this review.

To establish a consensus regarding the origin of myofibroblasts, Driskell and colleagues used lineage tracing and reported two distinct fibroblast populations in the upper and lower dermis [133]. Papillary dermal fibroblasts are responsible for hair growth and arrector pili muscle formation, while the reticular dermal fibroblasts play a significant role in ECM production [133]. Similarly, using the *Engrailed-1 (En1)* gene for embryonic lineage mapping, Jiang et al. [134] followed the fate of two subsequent populations of fibroblasts in a mice model of skin wound healing; *En-1* lineage-past fibroblast (EPFs) and *En1-*lineage-naive fibroblasts (ENFs). The authors reported a dynamic replacement of ENF progenitors by EPFs as the wound progressed and pointed to an increase in the expression of EPFs as a significant source of scarring. Besides *Engrailed-1,* the transcription factor PU.1 was reported to control tissue fibrosis [134]. Genetic ablation of PU.1 expressing gene *SPI1,* using CRISPR–Cas9 system, resulted in lower collagen production, but the expression of α-smooth muscle actin (α-SMA) and F-actin were not changed. However, overexpression of PU.1 in fibroblasts induced a profibrotic phenotype characterized by increased collagen, α-SMA, and F-actin production. Inhibition of PU.1 by an anti-fibrotic pharmacological mediator, DB1976, prevented skin fibrosis [135]. Although the role of fibroblasts in tissue repair is widely accepted, debate continues about their specific characterization. Robust characterization of fibroblasts based on surface protein expression, functional roles, and tissue niche will aid in developing novel treatments for fibrotic disorders.

### 4.6. Role of the Fascia in Wound Closure and Fibrosis

Current investigations are revealing a unique role for the subcutaneous fascia in wound closure and scarring. Utilizing fate mapping and live imaging, researchers traced the rise of embryonic Engrailed-1 positive fibroblasts from the fascia to the wound bed and subsequently to the skin surface [136]. Blood vessels, macrophages, and peripheral nerves are embedded in this raised jelly-like matrix, which may explain the morbidities of itch and pain emanating from some scars. This is a new line of investigation that may be worth pursuing to gain better insights into the pathophysiology of fibrosis.

## 5. Remodeling the Wound

The remodeling phase of wound healing starts by the end of the proliferation phase where wound reepithelization through keratinocytes and ECM deposition by the fibroblasts and endothelial cells occurs. In normal wounds, this phase lasts weeks to months and is characterized by wound contraction and scar maturation. In burns, the remodeling phase is protracted due to prolonged inflammation as detailed above.

During the remodeling phase of wound healing, the skin/scar acquires an ultimate morphology that mostly depends on the final organization of collagen fibers. In normal scars, the collagen fibers are small in parallel bundles. In hypertrophic scars, the collagen fibers are thin, more abundant and cross-linked [137]. During the remodeling phase, myofibroblasts also secrete Decorin; a protein that regulates collagen fibrogenesis by presenting as a “C” shape localized between the collagen fibrils to assure a uniform spatial fibril arrangement [138]. The fate of myofibroblasts during remodeling determines whether the wound closes and continues to develop a hypertrophic scar. In non-hypertrophic scars, myofibroblasts surrounded by fibrillar collagen may cause adverse effects leading to cell cycle arrest [139] or loss in the ability to adhere and thus undergo apoptosis [3].

As mentioned above, mechanical tension and increased inflammatory cytokines concentration, like TGF-β, drive fibroblast differentiation into myofibroblasts by the end of the granulation phase [137]. Myofibroblasts express high levels of α-smooth muscle actin (SMA), stress fibers, and contribute significantly to wound contraction [140]. Myofibroblasts also produce substantially more collagen than their regular counterparts. Collagen III in the ECM is replaced by collagen I, which has higher tensile strength but takes longer to deposit. Collagen organization is also altered in hypertrophic scars, and the healed skin can only achieve ~80% of the original tensile strength [141]. In burns, excessive and prolonged inflammation leads to excessive pathologic collagen deposition and fibrosis. Therefore, the attenuation of the inflammatory response can minimize aberrant collagen production.

### 5.1. Myofibroblasts and Apoptosis

During the wound healing process, skin fibroblasts that expanded in the proliferation phase now acquire a contractile phenotype by expressing high levels of the motile α-SMA protein, which aids fibroblast migration and ultimate wound closure. These fibroblasts, now termed myofibroblasts, were first described by Gabbiani et al. [131] and participate in the wound healing process by depositing substantial amounts of ECM proteins including collagen, elastin, and hyaluronic acid. Myofibroblasts are activated by inflammatory cytokines. TGF-β acts as the critical cytokine in epithelial−mesenchymal transition (EMT) and myofibroblast differentiation. In healthy wound healing, myofibroblast populations should dissipate when the wound is closed, mainly through apoptosis, or revert to quiescent fibroblasts [137,142]. In fibrotic conditions, myofibroblast apoptosis is delayed and the cells continue to express collagen and other ECM components. In burns, the prolonged inflammatory response over-activates myofibroblasts leading to overexpression of various components of the ECM and, therefore, the development of hypertrophic scars. Recent studies have targeted fibrosis by inducing myofibroblast apoptosis [143,144] including the use of gene therapy [144] and cuprous oxide nanoparticles [145].

New research has focused on ways to accelerate myofibroblast apoptosis or inhibit the transformation of fibroblasts into myofibroblasts [146]. The anti-fibrotic drug pirfenidone reduces the profibrotic and contractile phenotype of differentiated human dermal myofibroblasts [146]. Pirfenidone is a pyridine (5-methyl-1-phenyl-2-(1H)-pyridone) that is FDA-approved for the treatment of idiopathic pulmonary fibrosis [147,148]. Pirfenidone reduces hypertrophic scarring following a burn, animal bites, and in diabetic ulcer wounds [149,150,151,152] and a study by Wells and Leung [146] have shown that pirfenidone may reduce fibrosis through inhibition of fibroblast differentiation into myofibroblasts. Additional evidence has demonstrated that Pirfenidone can reduce the expression of αSMA and the contractile activity of fibroblasts while concurrently increasing the mRNA expression of MMP-1 [146].

New approaches are using regenerative therapies to reprogram myofibroblast differentiation in hypertrophic scarring [153,154]. Adipocytes have been shown to modulate myofibroblast differentiation through the activation of the bone morphogenetic protein -2 (BMP-2) and BMP-4 [154]. In fact, adipocytes initiate tissue remodeling by secreting BMP-4 to activate the signaling of nuclear receptor PPAR-y [153]. The role of PPAR in fibrosis and scarring is detailed further in Section 5.6 of this review.

### 5.2. Collagen Degradation and the Role of MMPs

Matrix metalloproteases (MMPs) are endopeptidases using calcium or zinc in their active sites. They are involved in the degradation of collagen and other ECM components. MMPs are grouped into four categories: collagenases, gelatinases, stromelysins, and membrane-type MMPs. The collagenases include MMP-1, MMP-2, MMP-8, and MMP-13. MMPs are essential regulators of collagen degradation that have been shown to cleave collagen I and III in scar tissue. MMP activation may reduce excessive collagen deposition and subsequent scarring. However, over-activation of MMPs may impede wound closure, increase the risk of infection, and further prolong the inflammatory phase.

Various studies have shown that MMPs modulate skin regeneration and hypertrophic scarring post-burn. Clinically, plasma levels of both MMP-2 and MMP-9 were significantly increased in patients that received a dermal regeneration Integra graft [155]. Using the Red Duroc pig model of hypertrophic scarring, DeBruler and colleagues [156] have shown that MMP-2 protein expression peaked four weeks after burn and subsequent split-thickness autographs. Using the same model, others have shown that MMPs are differentially regulated in response to compression therapy, with MMP-7 being the most downregulated [157]. Pharmacological approaches have been used to suppress collagen production by increasing MMPs as well [158]. The anti-inflammatory drug methotrexate reduces MMP-1 through the MAPK pathway [158], and kynurenine was also shown to increase MMP-1 and -3 expressions to improve wound healing in vivo through the same pathway [159]. MMP activity is also regulated by the tissue inhibitors of metalloproteinase (TIMPs). TIMP-1 and MMP-1 proteins are highly expressed in patients with extensive post-burn hypertrophic scars [160].

### 5.3. Role of the Ubiquitin-Proteasome System in Fibrosis

Various studies have addressed the role of ubiquitination and deubiquitination in idiopathic pulmonary fibrosis [161] as reviewed by Li et al. [162]. However, the role of this pathway in myofibroblast differentiation during scar formation is not well understood. We have reported that the basal levels of β2-AR ubiquitination were higher in hypertrophic scar fibroblasts from burn patients compared to non-burn skin fibroblasts, suggesting accelerated receptor degradation [163]. Recent studies have elucidated the role of the ubiquitination in both normal tissue repair and fibrosis and identified specific ubiquitin ligases in this process. Tetratricopeptide repeat domain 3 (TTC3), a new ubiquitin E3 ligase, positively regulates TGF-β_1_-induced epithelial to mesenchymal transition and myofibroblast differentiation [164]. TTC3 induces the ubiquitination and proteasome-dependent degradation of the Smad ubiquitination regulatory factor 2 (SMURF2) [164]. This leads to a suppression of SMAD2/3, which in turn activates TTC3 [164]. SMURF2, mainly localized in the nucleus, binds to SMAD7, translocates out of the nucleus, and induces the turnover of TGF-β receptors [165]. Our group has previously shown that the increased expression of SMURF2 is involved in the progression of hypertrophic scarring after burn [166]. Modulation of another ubiquitin 3 ligase, Mitsugumin 53 or MG53, was shown to be essential to the regulation of wound healing and scarring [167]. MG53 is a member of the tripartite-motif (TRIM) family of proteins that plays an essential role in the cell membrane repair machinery. MG53 deficient mice with excisional wounds display delayed wound healing and abnormal scarring and significant defects in skin architecture and collagen overproduction [167].

### 5.4. The Role of Plasminogen Activation Inhibitors and MicroRNAs in Wound Healing and Scarring

Recent studies have pointed to new players that can reduce HS if targeted at the right time. Among those is plasminogen activator inhibitor-1 (PAI-1). PAI-1 is a serine protease inhibitor that functions as the principal inhibitor of the tissue plasminogen activator (tPA) and urokinase (uPA) [168], the activators of plasminogen, and hence controls fibrinolysis. PAI-1 protein expression is increased in cancer [169], obesity [170], and metabolic syndrome [171,172]. Increased levels of PAI-1 can lead to an increased risk of thrombosis and atherosclerosis [172]. PAI-1 controls tissue homeostasis by regulating the proteolytic activities of uPA/tPA/plasmin/MMP [173], therefore regulating the degradation of collagen and other ECM components [173]. A very recent report showed that PAI-1 regulates fibrosis by mediating the effects of mast cells on fibroblasts, therefore regulating collagen secretion by these cells [174]. These data also connect with and underline the important role of the immune response in the regulation of fibrosis.

The role of non-coding RNAs, including miRNA and long non-coding RNAs (lncRNAs) in the regulation of wound healing and scarring, is well-established and was extensively reviewed by Herter and Landén [175]. PAI-1 also seems to drive collagen degradation through microRNAs. In fact, Collagen I generation in hypertrophic scars is regulated by MiR-10 and MiR-181C targeting of PAI-1 and uPA, respectively [176]. Pang et al. [177] have shown that miR-152-p reduces keloid scar fibroblast proliferation and migration and promotes apoptosis through the Smad2/3 pathway. Similarly, MiR-23b inhibits the proliferation and migration of heat-denatured fibroblasts by directly targeting Smad3 and Notch1 signaling pathways [178]. MiR-181b reduces the expression of Decorin by dermal fibroblasts, and its inhibition increases Decorin production, destabilizes the formation of collagen bundles, and prevents scarring [179]. The same study identified MiR-181b as a potential therapeutic target to reduce fibrosis [179]. MiR-148b also targets TGF-β signaling to regulate EMT and angiogenesis during wound healing [180]. Together, these studies open new avenues for the therapeutic application of miRNAs in tissue repair, acceleration of wound healing, and reduction of scarring.

Besides miRNAs, long non-coding RNAs (lncRNAs) are also involved in wound healing and scarring by modulating TGF-β-induced fibrosis [181,182]. LncRNAs are a class of RNAs longer than 200 nucleotides without protein-coding capacity. LncRNAs are involved in the pathophysiology and development of human hypertrophic scars, and high throughput screening has shown that lncRNAs are differentially expressed in human hypertrophic scars [183]. A microarray study showed that 6104 lncRNAs and 2952 mRNAs were differentially expressed in hypertrophic scars compared to normal fibroblasts [182]. Among the lncRNA, expression of AC067945.2 was shown to decrease in hypertrophic scar tissues [184]. Overexpression of AC067945.2 slightly stimulated early apoptosis in normal skin fibroblasts without affecting cell proliferation [184]. Co-expression analysis has shown that the expression of lncRNAs NR_125715 and NR_046402 in hypertrophic scar fibroblasts correlated with TGF‑β2 and POLD1 mRNA expression [182]. LncRNA8975-1 is increased in HS fibroblasts and regulates collagen production [185]. The same group identified 1871 lncRNAs that are differentially expressed between regressive and mature scars including lncRNA8975-1, AC097662.2, and RP11-586K2.1 [185].

### 5.5. Epigenetic Regulation of Wound Healing

Persistent expression of pro-fibrotic genes after wound resolution results in fibrosis [186], and accumulating evidence suggests a role for epigenetics in this process [187]. Various studies have demonstrated that pathologically profibrotic fibroblasts are epigenetically altered [188,189,190]. Epigenetics is one of the most reliable mechanisms to transfer the gene function from mother to daughter cells without any changes in the DNA sequences. This process is carried out in two different ways via DNA methylation or histone modifications. Aberrant hypermethylation of certain genes facilitates profibrotic gene activation and drives fibrogenesis. Fibroblasts from scars and keloids express high levels of DNA methyltransferase 1 (DNMT1) compared to normal skin fibroblasts [76]. Following treatment with a methylase inhibitor, 5-aza-2-deoxycytidine, the expression of DNMT1, and TGF-β were decreased while Smad7 mRNA transcript levels and apoptosis markers were increased in the hypertrophic scar fibroblasts [76].

Histone methylation and acetylation are both implicated in profibrotic diseases. Histone deacetylases are upregulated in normal and keloid scars [190]. Inhibition of histone deacetylation, using trichostatin A (TSA), blocks TGF-b mediated myofibroblast differentiation [191] and collagen expression in scar fibroblasts [189]. TSA was also shown to reduce hypertrophic scarring in the rabbit ear excision model [192]. Together, these data suggest a potential therapeutic application of histone deacetylase inhibitors in the reduction of fibrosis and scarring.

Histone methylation is mediated by methyltransferases and demethylases, and its effects on gene activation or repression depend on the extent of methylation and position of this modification in the histone proteins [193]. Historically, activation of gene expression is linked to trimethylation of histone 3 at the lysine 4 (H3K4me3) and dimethylation or tri-methylation at lysine 79 of histone 3 (H3K79me2/3) [194]. Gene repression, however, is linked to trimethylation at lysine 9 (H3K9me3), lysine 27 (H3K27me3), and lysine 20 (H3K20me3) [194]. The role of histone methylation in fibrotic diseases is well-established [195]. In the skin, expression of H3K27 histone demethylases is the most upregulated during wound repair [196]. TSA and 5-aza-deoxycytidine were shown to reverse epigenetic repression of the Fli1 gene [197], a gene involved in the regulation of collagen expression [198], and to reduce collagen expression in scleroderma fibroblasts [197]. A time-course scarring study of engrailed positive fibroblasts (EPFs), a precursor of activated fibroblasts, demonstrated that transposase-accessible chromatin sequencing (ATAC-seq) is functionally distinct in these fibroblasts and expresses major differences in genes associated with fibrosis [199]. These findings strongly support a role of epigenetic regulation of gene expression in the fibroblast phenotype and open the doors for novel potential therapeutic targets.

### 5.6. Transcriptional Regulation of Myofibroblasts Differentiation, Contraction, and Wound Healing

Bellavia et al. [200] extensively reviewed the role of transcription factors in wound healing and re-epithelization. Many transcription factors were shown to play a role in wound healing and scarring. That includes the activator protein-1 (AP-1) transcription factor family members including Jun, Fos, and ATF dimers and Calveolins [201]. The myocardin-related transcription factors (MRTFs) regulate myofibroblasts’ contractility by associating with serum response factor (SRF) and activating genes implicated in cytoskeletal dynamics [129]. The Hippo members Yes-associated protein 1 (YAP1) and transcriptional coactivator with PDZ-binding motif (TAZ) are activated by tissue stiffness and TGF-β1 [202]. Knockdown of YAP-1 abrogates TGF-β-induced expression of SMA stress fibers and collagen type I deposition [202]. In a full-thickness, skin wound model, knockdown of YAP/TAZ delays wound closure and modulates the expression of various components of TGF-β signaling including Smad-2, p21, and Smad-7 [164]. While some data suggest that Hippo member transcription factors YAP-1/TAZ contribute to the contractile phenotype of myofibroblasts in the full-thickness burn model, their role in scar development after burn is less understood. The transcriptional modulation of metabolic reprogramming also regulates the contractile phenotype of myofibroblasts as shown by genetic and pharmacologic approaches that aimed to impair mitochondrial biogenesis or glycolysis [203].

Nuclear receptors also play a role in wound healing. Among those, the estrogen receptors (ERs) contribute to cutaneous aging, delayed or impaired wound healing, and increased risk of infection [204,205]. Decreased estrogen levels with age reduce both types I and type III collagen and shift the collagen III/I ratio in the skin [206]. Estrogen receptors are involved in all three phases of wound healing; they regulate the inflammation phase by modulating the infiltration of immune cells to the wound bed [207]. Most immune cells including macrophages, monocytes, neutrophils, dendritic cells, and mast cells express ERs, further implicating these nuclear receptors in regulating the inflammatory phase of wound healing [207,208,209,210]. More importantly, estrogens were shown to alter macrophage polarization between a pro-inflammatory M1 to an anti-inflammatory M2 phenotype [211,212]. ERs are also expressed in fibroblasts, endothelial, epithelial cells, and keratinocytes, therefore modulating the proliferative phase of wound healing [207]. By reducing inflammation and modulating the proliferation phase of wound healing, ERs contribute to accelerated wound closure. Simultaneously, ERs reduce aberrant collagen deposition and subsequent scaring by inhibiting TGF-beta signaling by accelerating the degradation of Smad 2/3 [213].

Another group of nuclear receptors that regulate wound healing is the peroxisome proliferator-activated receptors (PPARs). The role of PPARs in skin wound healing was extensively reviewed by Yin and Smith [214]. Skin injury stimulates the expression of PPAR-α and PPAR-β the site of the wound [215,216] and regulates wound healing by antagonizing the effects of TGF-β and TNF-α [215]. Expression of PPAR-γ is also induced by skin injury [216] and is known to suppress the profibrogenic response to injury by antagonizing TGF-beta signaling [217]. PPAR-γ is usually bound to the retinoid X receptor (RXR) and its corepressors. After receptor activation, the PPAR-γ RXR complex detaches from the repressors and binds the PPAR-γ response elements to activate gene expression [218]. PPAR-γ reduces fibroproliferative responses by competing with Smad 2/3 for its cofactor p300 [219]. These effects are not observed in the PPAR-γ knockout mice, which exhibit a profibrotic skin phenotype [219].

## 6. Clinical Trials to Reduce Fibrosis

With the skyrocketing increase in the economic burden of fibrosis and skin-related diseases, there has been a significant increase in the number of clinical trials aiming to treat fibrotic-related morbidities, specifically the ones related to lung and liver fibrosis [220,221]. Currently, there are no FDA-approved treatments for skin-related fibrosis. According to clinicaltrials.gov, 43 registered interventional clinical trials were primarily funded by industries in the last 10 years. These interventional studies include biological mediators like EXC 001 [222], an antisense oligonucleotide that targets connective tissue growth factor, tilapia skin for surgical wound dressing [222], fat lipofilling [223], and stem cell injection [224]. Other trials have used pharmacological mediators such as PF-06473871 [225] and RXI-109 [226] to ameliorate hypertrophic scars and P144 to treat skin fibrosis by blocking the interaction between TGF-β1 type III and TGF-β1 receptors (Acces on26^th^ Nov, 2019, https://clinicaltrials.gov/ct2/show/NCT00574613).

Although TGF-β is regarded as the master regulator of fibrosis [227], clinical trials targeting the TGF-β signaling pathway have yet to produce a therapeutically beneficial outcome in treating fibrosis-related morbidities. Using anti-TGF-β antibodies, two trials, CAT-152 using a dose of 100 micrograms in 100 microliters (TGF-β2 > TGF-β3, 2002) [228] and CAT-192 using a dose of 5 mg/kg (TGF-β1, 2007) [229], failed to show promising results in treating patients with conjunctival fibrosis undergoing trabeculectomy and cutaneous SSc, respectively. Unfortunately, four deaths and significant adverse side effects were reported in the study using CAT-192, compared to the placebo [229]. Besides TGF-β signaling, physical interventions like non-ablative fractional laser therapy [223], gel-creams, and silicone garment therapy [230] for preventing hypertrophic scarring have also been reported.

## 7. Molecular Modulation of Hypertrophic Scarring Following Severe Burns

Post-burn hypertrophic scars are the epitome of wound healing phases in disarray. These scars are serious complications of deep thermal burns and remain the biggest challenge to patient recovery [231]. Burn survivors’ scars are raised, pruritic, prevent normal function, and severely reduce the quality of life. Traditionally, hypertrophic scarring is assessed using various scales including the Vancouver Scar Scale (VSS) or the modified patient and observer scar assessment scale (POSAS) [232]. Recent methods for scar assessment include the use of a three-dimensional camera [233]. Three-dimensional stereo-photogrammetry was recently introduced as a reliable method for assessing scar volume in clinics [234]. The location of scars on the face and other visible areas affects the patient self-esteem and prevents total reintegration into society [235,236]. While the incidence of hypertrophic scars is high following deep thermal injury, it is difficult to predict which patients will scar and those that will not. Equivalently, patients respond differently to similar therapeutic approaches, warranting the need to understand mechanisms underlying hypertrophic scarring after burn and the differential response to treatments.

Various types of surgical and non-surgical strategies are used for the treatment of hypertrophic scars, including the application of dynamic and static mechanical forces, pressure garments [237], and light-based therapies including the CO_2_ ablative fractional laser [238,239]. Administration of various injectable and topical treatments including corticosteroids, immune modulators, hyaluronidases, and cell-based therapies [240,241,242,243] have demonstrated limited efficacy. Injection of uncultured centrifuged adipose tissue in burn survivors has resolved pain, restored movement, and significantly reduced scar hypertrophy [244]. The fat tissue in the above study was harvested by liposuction using the Coleman technique to minimize tissue processing and contains adipocytes, SVF, and blood cells. However, none of these treatments are entirely effective in fully attenuating hypertrophic scars and associated morbidities, making surgical revisions a last resort for these patients. All evidence further supports the introduction of novel research initiatives to prevent scar development and progression.

### Conclusions

Skin fibrosis and scarring involve an imbalance of various mediators throughout wound healing. Efficacious therapies should include a multidimensional approach targeting all three phases of wound healing, starting by moderating the inflammatory response, limiting the excessive proliferation of myeloid cells and myofibroblasts, and applying physical and biological interventions during wound remodeling.

## Figures and Tables

**Table 1:** Difference between M1 and M2 macrophages.

Profile	M1	M2
Temporal appearance in the wound	Towards the middle of the inflammation phase	Towards the end of the inflammation phase
Activated by	IFN-γ, LPS, GM-CSF	IL-4, IL-10, IL-13, TGF-β
Secretory profile	IL-1β, IL-12, IL-18 and TNF-α, iNOS	High IL-10, Arginase
Surface markers	MHC-II, CD68, CD80	CD206, CD163+CMAF
Physiological roles	Active phagocytic and microbicidal phenotype	Anti-inflammatory
Over-activation	Tissue destruction	Remodeling and excessive collagen production

## Abbreviations

AIM2	absent in melanoma 2
AP-1	activator protein-1
BMP	bone morphogenetic protein
DAMPs	danger-associated molecular patterns
DNMT1	DNA methyltransferase 1
ECM	extracellular matrix
EMT	epithelial−mesenchymal transition
En1	Engrailed-1
EndMT	endothelial cells to mesenchymal cells
ERs	estrogen receptor
Fli1	Friend leukemia virus integration 1
HIF-1	hypoxia-inducible factor-1
HMGB1	high-mobility group box-1
HS	hypertrophic scars
IL-1β	initiators interleukin-1beta
IL-4Rα	interleukin-4 receptor α
LC	Langerhans cells
LH2	lysyl hydroxylase 2
lncRNAs	long non-coding RNAs
LRG-1	leucine-rich-alpha-2-glycoprotein 1
miRNA	microRNA
MMP	matrix metalloprotease
MRTFs	myocardin-related transcription factors
NETs	neutrophil extracellular traps
NK	natural killer
PAI-1	plasminogen activator inhibitor-1
POSAS	patient and observer scar assessment scale
PPAR	peroxisome proliferator-activated receptor
PRP	platelet-rich plasma
PRRs	pattern-recognition receptors
RAGE	receptor for advanced glycation end
Rv	resolvins
RXR	retinoid X receptor
SMURF	Smad ubiquitination regulatory factor 2
SSc	systemic skin sclerosis
TAZ	transcriptional coactivator with PDZ-binding motif
TGF-β	transforming growth factor-beta
TIMP	tissue inhibitors of metalloproteinase
TMF	trimethoxy-isoflavone
TNF-α	tumor necrosis factor-alpha
TRIM	tripartite-motif
TSA	trichostatin A
TTC3	tetratricopeptide repeat domain 3
VSS	Vancouver Scar Scale
YAP1	Yes-associated protein 1
α-SMA	α-smooth muscle actin
